# NIA *Caenorhabditis* Intervention Testing Program: identification of robust and reproducible pharmacological interventions that promote longevity across experimentally accessible, genetically diverse populations

**DOI:** 10.1007/s11357-025-01627-4

**Published:** 2025-04-03

**Authors:** Monica Driscoll, Christine A. Sedore, Brian Onken, Anna L. Coleman-Hulbert, Erik Johnson, Patrick C. Phillips, Gordon Lithgow

**Affiliations:** 1 Rutgers, The State University of New Jersey, Piscataway, NJ USA; 2 University of Oregon, Eugene, USA; 3 Buck Institute for Research on Aging, Novato, CA USA

**Keywords:** *C. elegans*, Genetic model, Pharmacological interventions, Pro-longevity, Healthy aging, Genetic diversity, Reproducibility

## Abstract

A core facet of the National Institute on Aging’s mission is to identify pharmacological interventions that can promote human healthy aging and long life. As part of the comprehensive effort toward that goal, the NIA Division of Biology of Aging established the *Caenorhabditis* Intervention Testing Program (CITP) in 2013. The *C. elegans* model (with an ~ 21 day lifespan) has led the field in dissection of longevity genetics and offers features that allow for relatively rapid testing and for the potential elaboration of biological mechanisms engaged by candidate geroprotectants. CITP builds on this foundation by utilizing a genetically diverse set of intervention test strains so that “subjects” represent genetic diversity akin to that that between mouse and humans. Another distinctive aspect of the CITP is a dedicated focus on reproducibility of longevity outcomes as labs at three independent test sites confirm positive outcomes. The overall goal of the *Caenorhabditis* Intervention Testing Program (CITP) is to identify robust and reproducible pro-longevity interventions affecting genetically diverse cohorts in the *Caenorhabditis* genus. A strong Data Collection Center supports data collection and dissemination. Pharmacological interventions tested by CITP can be nominated by the general public, directed by in-house screens, or supported by published scientific literature. As of December 2024, CITP tested > 75 compounds and conducted > 725,000 animal assays over 891 trials. We identified 12 compounds that confer a ≥ 20% increase in median lifespan to reproducibly and robustly extend lifespan across multiple strains and labs. Five of these interventions have pro-longevity impact reported in the mouse literature (most CITP positive interventions are not tested yet in mouse). As part of the celebration of the 50th Anniversary of the NIA, we review the development history and accomplishments of the CITP program, and we comment on translation and the promise of advancing understanding of fundamental aging biology that includes the pharmacological intervention/health interface.

## Introduction

Geroscience combines the study of the biology of aging with the understanding of the molecular and cellular changes underlying the biology of age-related diseases. The core notion is that aging is the primary risk factor of chronic disease of the aged. The geroscience approach is based on targeting the drivers of aging to delay the onset and/or mitigate the severity of morbidities associated with older age [1]. In addition, older adults often present with multiple comorbidities that traditionally have been treated with multiple pharmaceuticals. The geroscience approach of targeting aging suggests that many diseases could be mitigated by a small number of therapeutic approaches. Developing safe interventions that target the molecular and cellular drivers of aging (geroprotectants) will ultimately result in optimized care for older adults and address the limitations and risks of polypharmacy.

Besides exercise and dietary interventions, which have been extensively studied and proven to be efficacious in delaying the onset of aging phenotypes and disease, one attractive option for addressing aging challenges is the identification of pharmacological interventions that would delay the onset of aging and therefore possibly delay and/or reduce the severity of chronic and age-related diseases. A precedent for such an effect is observed by caloric restriction of rodents leading to a reduction in all-cause mortality. Screening for compounds that can target aging is challenging due to both the complexity of the aging phenotype and the genetic heterogeneity of the human population. The ideal model system for a geroprotectant screening effort is one that is short-lived, cost-effective, amenable to automation, genetically diverse, and translatable. The invertebrate *Caenorhabditis* displays those characteristics: this nematode has been adopted to study fundamental physiological processes common to all animals, including aging [2], and for extensive drug discovery [3–6]. Of note, 60–80% of *C. elegans* genes have human homologs [4], including 66% of human genes associated with disease, supporting the striking conservation of fundamental biological mechanisms from invertebrates to humans. Indeed, the investigation of aging genetics in *Caenorhabditis* has revealed highly conserved biological pathways and mechanisms of import for nematode and mammalian aging [7] (e.g., insulin-signaling pathway, innate immunity regulation, protein homeostasis).

Several approaches have led to the establishment of credible candidates for interventions in human aging. FDA-approved drugs such as rapamycin and metformin have been identified through a combination of laboratory studies and human clinical data. Others, such as senolytics, target mechanisms established in mammalian cell culture and then tested in vivo. Computational predictions based mainly on genetic data are also being applied.

We posit that extension of metazoan lifespan remains a critical first step in identifying strong candidates for aging interventions. Very few species can be used for mid-to-high throughput screening of drug-like candidates for lifespan. The short generation time and lifespan of *Caenorhabditis* strains (2.5–3.0 days/generation; ~ 21 day lifespan) facilitate compound screening in a few weeks. More than 100,000 drug-like compounds, metabolites, and natural products have been screened directly for effects on lifespan. Furthermore, recent technologies such as imaging and artificial intelligence make it possible to automate mid-to-high throughput drug screens (Munkácsy and Pickering (2021). In: *Handbook of the Biology of Aging*. Elsevier, Amsterdam, pp 199–217) [8].

The *Caenorhabditis* Intervention Testing Program (CITP) was promoted by the Division of Aging Biology (DAB) at the National Institute on Aging (NIA) and started in 2013. The CITP was modeled on the example of the successful mouse Interventions Testing Program (ITP) (Nadon et al. (2016). In: *Handbook of the Biology of Aging*. Elsevier, Amsterdam, pp 287–303) [9] and was intended to address the testing bottleneck caused by lengthy and costly mouse lifespan experimentation. Thus, the choice of *Caenorhabditis* was anticipated to allow for higher throughput testing, enhanced genetic diversity within test sets, and deeper understanding of biological mechanisms impacted by candidate geroprotectants.

One core element of both ITP and CITP was the independent experimental replication in three laboratories at three institutions to address the reproducibility gap that concerns some high profile publications, including of aging studies. In the backdrop of the debate on irreproducibility in psychological and biological sciences, the ITP and CITP was a robust response by the NIA to ensure the foundation of future pre-clinical and clinical research in aging.

**Fig. 1:**
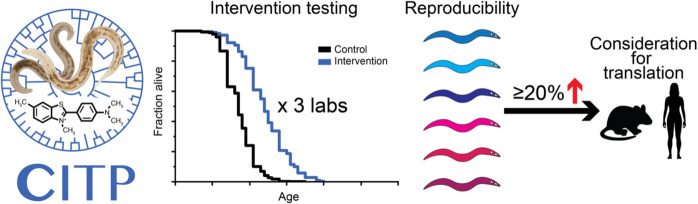
Overview of the core CITP mission. The *Caenorhabditis* Intervention Testing Program measures longevity outcomes of candidate pharmacological interventions in a genetically diverse test set of *Caenorhabditis* strains. Data are replicated at three independent sites, with data processing and sharing via a Data Coordination Center. Interventions can be nominated from outside scientists, selected from in house library screens, or inspired by scientific literature

The goal of the CITP is to test, under standardized conditions and in a reproducible and robust manner, candidate pharmacological interventions for their capacity to extend lifespan and/or healthspan across genetically diverse species and/or strains of *Caenorhabditis* (Fig. 1). The CITP goals are achieved through a consortium of three academic research laboratories (led by principal investigators Monica Driscoll at Rutgers University, Gordon J. Lithgow at the Buck Institute for Research on Aging, and Patrick Phillips at the University of Oregon) working in close collaboration to select candidate compounds and *Caenorhabditis* species/strains and to implement common protocols, pipelines, and standard operating procedures. These efforts have been established by consensus of a Steering Committee consisting of the principal investigators and at least four biogerontology subject matter experts. Patrick Phillips is also the principal investigator of the CITP Data Coordinating Center that manages data collection and storage, conducts bioinformatic and statistical analysis, and organizes the public CITP Data Portal. The CITP and Data Coordinating Center are funded by the NIA through four cooperative agreement grants. The compounds that enter the CITP testing pipeline are prioritized by the Steering Committee from a list that includes nominations from the larger scientific community as well as from in-house CITP compound library screens and from literature assessment.

The scope of the CITP has been refined over time as data on several CITP-tested compounds in different nematode species accumulated, and our understanding of the biology of aging, and its interface with pharmacological intervention, deepened. Similarly, the testing pipeline has gone through a few iterations and improvements that both reflect capacity for increased throughput and modernized options for candidate intervention assessment. In addition to compounds nominated by the community, in-house compound library screens have identified new candidates that enter the CITP pipeline. Currently, the CITP uses lifespan and locomotory health as criteria for compound efficacy. Transcriptomic profiles are generated for compounds showing ≥ 20% increase in lifespan to gain insight into compound molecular targets and mechanisms, potential phylogenetic conserved pathways, and genetic contributions to intervention responses. All CITP results are published in peer-reviewed scientific journals, irrespective of whether the effects (robust or not) show an increase, decrease, or no impact on lifespan or health span.

The CITP has advanced considerably since its inception in 2013. CITP has generated large, consistent and reproducible datasets that can be easily interrogated to allow for comparison of intervention performance under the same experimental conditions. Data has been analyzed to determine within-laboratory and between-laboratory reproducibility of lifespan extension and has anchored development of statistical methods for lifespan, locomotory status, and mortality analyses. Here, we describe the CITP workflow optimization and the efforts required to attain and assess reproducibility, challenges and approaches to intervention identification, and the accomplishments of CITP, both expected and unexpected. We propose observations relevant to the goal of translation as well as future perspectives for the CITP and the NIA efforts to advance toward the identification of effective geroprotectants.

## CITP workflow overview

**Fig. 2:**
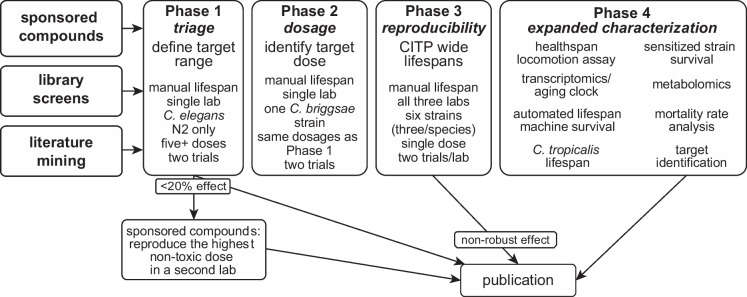
Summary of the CITP 5.2 workflow, January 2025. Test compounds enter the pipeline via three routes: (1) nomination from the field with applications ranked and approved by the CITP Access Panel and Steering Committee (see CITP Intervention Nomination Guide at the NIA website); (2) positive outcomes of CITP library screens currently focused on induced thermotolerance testing; (3) selection based on the scientific literature (documented longevity engagement, machine learning, etc.). In phase 1, compounds are first tested in *C. elegans* reference strain N2 at five dosages in two trials at one CITP site. Sponsored compounds that meet the ≥ 20% mean lifespan standard are re-tested at a second CITP site. All compounds that confer ≥ 20% increase in mean lifespan move into phase 2 in which a single CITP lab tests effects in *C. briggsae* strain AF16 at five dosages in two trials. Candidate compounds then enter phase 3 testing in which all three CITP labs conduct manual lifespan survival assays in three *C. elegans* and three *C. briggsae* strains, two trials per lab, at the optimal dose identified in phases 1/2. Compounds that reproducibly confer ≥ 20% lifespan extension in multiple strains progress to expanded characterization in phase 4 in which swimming capacity in old age is evaluated, and a combination of testing in three *C. tropicalis* strains, transcriptomic analysis (including aging clock assessment), metabolomic study, determination of mortality rates, potential target identification, automated lifespan machine survival assays, and testing of compound efficacy in longevity mutant backgrounds (assay constellation is dictated in part by extent of survival enhancement and consideration of current understanding of the compound’s mechanism). All test outcomes are published regardless of impact on longevity, with phase 4 data informing on healthspan and suggesting hypotheses for mechanism of action for most impactful compounds

When the CITP initiated intervention testing, we performed manual survival determinations of compound effect on three *C. elegans*, three *C. briggsae*, and three *C. tropicalis* strains at all three test sites [10]. Over time, we examined initial screen findings to modify the CITP pipeline with an eye toward enhancing the number of compounds that we could test annually. The current CITP workflow (version 5.2, Fig. 2) reflects streamlining to enhance throughput and expanding the basic characterization options for compound impact on animal health and lifespan for treatments that are found to extend longevity in multiple strains across the three CITP testing labs. Critically, we have introduced a cut off of ≥ 20% relative increase in survival threshold as a criterion for moving an intervention forward through the program. Compounds that do not increase lifespan by 20% or more in initial experiments are excluded from further analysis. This cutoff is designed to focus resources on compounds that are potent and can be most easily studied for mechanism. We note that our approach does not mean to diminish the potentially large biological significance of compounds that do not meet the criteria we set but rather is to maximize attention on the most potent compounds.

The current CITP workflow is summarized in Fig. 2. Efficacious compounds are routed through four stages of testing. In brief, compounds entering the pipeline are initially tested over a five-dose range on *C. elegans* genomic reference strain N2 at one site (phase 1; compounds that enter via the Access Panel applications are also tested at a second site to confirm outcome). For interventions that confer ≥ 20% median lifespan extension in *C. elegans* N2, a similar dose range is tested in representative *C. briggsae* strain AF16 (phase 2) to identify a target dose for testing across multiple strains. In phase 3, compounds that confer ≥ 20% median survival increase are assessed in manual survival assays using the most effective compound dose identified in phases 1/2. Phase 3 testing is on three *C. elegans* and three *C. briggsae* strains at all three CITP sites, with two independent trials/site.

Following statistical evaluation of outcomes (see below), compounds that reproducibly confer ≥ 20% median survival increase as compared to its untreated control strain (in multiple backgrounds) are advanced to the next stage of testing. Phase 4 features phenotypic, functional, and molecular analyses, including assessment of locomotory function at two older ages using CeleST (*C. elegans* swim test) [11]; survival analysis using automated lifespan machines [12], transcriptomic profiling and differential analysis in the presence and absence of compound (https://doi.org/10.7554/eLife.104375.1); and metabolomics, target identification, and testing effects in aging-compromised strains (for example, that lack the DAF-16/FOXO stress-responsive transcription factor). The extent of characterization is determined on a per-compound basis and is influenced by the degree of survival increase, known literature on the compound, practical concerns, and perceived level of public interest (for example, use as marketed health supplements).

### Considerations for compound selection: feeding the pipeline

The CITP seeks to identify interventions with the high potential to extend lifespan and/or delay the onset of disease and dysfunction using *Caenorhabditis.* To date, interventions have entered the CITP pipeline via multiple routes, all of which have identified pharmacological treatments that extend *Caenorhabditis* lifespan.

#### Peer-reviewed nominations from interested researchers who apply via the CITP Intervention Nomination process

CITP test compounds can be nominated by interested parties following the modalities described on the NIA-hosted CITP Intervention Nomination website (https://www.nia.nih.gov/research/dab/caenorhabditis-intervention-testing-program-citp/citp-intervention-nomination-guide). Compound nominators can be individuals, non-profit organizations, academic groups, or businesses. A requirement is that nominators must agree to disclosure of the molecular structure of a new compound tested by the CITP and to CITP publication of results, regardless of positive or negative outcome. Currently, applications are accepted and reviewed on an annual basis.

Applications involve documentation of rationale, preliminary data regarding potential geroprotectant effects, and information on compound chemical properties, availability, and cost. Given the challenges of precise replication of CITP data, CITP has moved away from testing interventions of undefined or variable composition (such as plant extracts). CITP does not test proprietary compounds that cannot be publicly identified immediately after study completion.

Applications are reviewed by members of an expert Access Panel (five scientists from the aging research community plus one investigator from each CITP site who is not the Principal Investigator). The top scored applications are presented to the CITP Steering Committee (made up of four outside experts who are researchers with a broad range of expertise across the aging field; the NIA Project Scientist and the three CITP Principal Investigators) who discuss and prioritize compound entry into testing.

For approved candidates, intervention testing is a collaborative process, and the compound nominator has access to all data as it is generated during the study. Compound sponsors can assist in data analysis and are offered to co-author resulting publications with the CITP.

The public entry path for CITP testing was initiated in 2021. The Access Panel and Steering Committee approved 9 of 12 applications for a total of 16 compounds entering the pipeline through this route. One of the first successful public-suggested compounds is sulforaphane (contained at high levels in broccoli), which can confer ~ 40% median lifespan extension (Sedore et. al., manuscript in preparation). A subset of other nominated compounds appear promising as they move through the current CITP testing pipeline.

#### Scientific literature mining and machine learning predictions

We initially focused mainly on compounds that were known to extend *C. elegans* lab reference strain N2 or compounds with high-profile attention in the anti-aging field, such as rapamycin [13] and metformin [14].

More recently, CITP is focusing on the intersection of datasets of compounds predicted to have the highest chance for engaging conserved aging pathways (doi: https://doi.org/10.1101/2024.10.23.619838). Predictions are based on the weighted intersection of three datasets: (1) compounds identified by drug/protein interactions to be likely to promote healthy aging transcriptional profiles [15]; (2) compounds identified by machine learning programs to have longevity promotion potential [16]; and (3) compounds identified to promote a “young” transcriptional signature [17]. We prioritized compounds that had not previously been tested for *C. elegans* longevity (to favor the identification of novel candidates), and we avoided testing compounds with known bactericidal activity (as these might limit the nematode food source to induce a dietary restriction response). This intersection/filtering strategy yielded 16 compounds that entered the CITP pipeline, 5 of which (all-trans-retinoic acid, berberine, fisetin, ritonavir, propranolol) significantly extended median lifespan (doi: https://doi.org/10.1101/2024.10.23.619838). The high “hit” rate of ~ 30% among these computationally predicted candidates underscores the power of trans-phyla computation combined with in vivo testing approaches to identify potential aging interventions. Furthermore, this approach provides a strong example of how CITP test data can help sort through and rank interventions that merit more detailed study across models. As artificial intelligence prediction power expands, the CITP is poised to rigorously test novel candidate interventions for efficacy in geroprotection.

#### Novel chemical library screens

To accelerate intervention discovery, CITP has also screened through selected chemical libraries for longevity and healthspan, work that is ongoing. In brief, high-throughput screens of two libraries (one containing compounds that target mitochondrial pathways and the other that features neuroprotective compounds) for compounds that confer enhanced survival or thermotolerance (long known to correlate well with longevity) [18] have yielded promising candidate interventions. Preliminary data suggest that novel molecules identified using this approach may exert positive outcomes in CITP testing.

In summary, the CITP casts a broad net of intervention consideration to maximize potential novel compound discovery. Multiple approaches toward efficacious intervention identification have proven successful in the CITP program.

### Basics of CITP operation and methods

#### The CITP reference strains mimic genetic diversity similar to that among mouse and humans

Most published studies of longevity-promoting compounds in *C. elegans* are conducted using the genetically homogeneous reference lab-adapted strain, designated as N2. Whereas genetic homogeneity offers myriad advantages to mechanistic dissection, genetic homogeneity is likely suboptimal for intervention discovery with translation potential. To more closely mimic a genetically heterogenous target population, the CITP established a representative test set of wild *Caenorhabditis* strains.

**Fig. 3:**
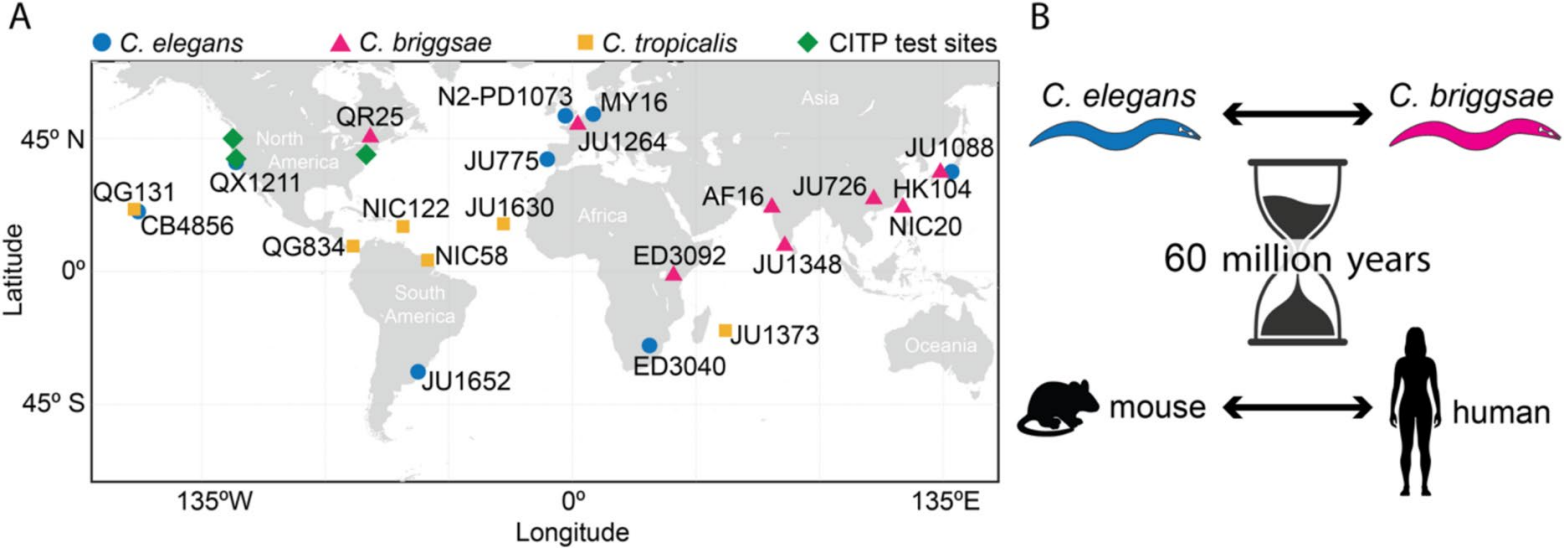
CITP wild strains have been collected from all over the world, and represent genetic diversity greater than mouse to human. **A** Geographic depiction of sites from which CITP references originated. Listed are the strain names, color coded to species as indicated. In green are the CITP lab sites. **B** Genetic diversity as measured by average genome-wide nucleotide diversity (π) between *C. elegans* and *C. briggsae*species is similar to that between mouse and human [19]

We initially chose to use 22 *Caenorhabditis* natural isolates spanning three species (*C. briggsae*, *C. elegans*, and *C. tropicalis,* each of which offers hermaphroditic reproduction advantages) for CITP analyses to maximize sampling of genetic and geographic diversity within each species [19] (Fig. 3). The genome sequences of most of these strains were available, which was a factor in their selection for the CITP study, and we filled in the remaining missing genome sequences [19].

The 22 nematode strains we selected from the three hermaphroditic species of *Caenorhabditis* were *C. elegans* (CB4856, ED3040, JU1088, JU1652, JU775, MY16, N2-PD1073 (a frozen ancestral reference line) and QX1211), *C. briggsae* (AF16, ED3092, HK104, JU1264, JU1348, JU726, NIC20 and QR25), and *C. tropicalis* (JU1373, JU1630, NIC122, NIC58, QG131 and QG834).

During the course of initial CITP studies, we learned about additional in-lab features of the original test strains (for example, avidity of burrowing deep into the agar), and we felt a need for increased throughput. We refined our workhorse test set to include three *C. elegans* (N2, MY16, JU775*)*, three *C. briggsae* (AF16, ED3092*,* HK104), and three *C. tropicalis* (JU1630, JU1373, QG834) strains. Over time, we have found *C. tropicalis* strains to be refractory to the majority of interventions we tested. Therefore, we further streamlined our core test pipeline to increase throughput by focusing first on *C. elegans* and *C. briggsae* responses; *C. tropicalis* is now tested in phase 4 for the most potent interventions we identify (Fig. 2).

We determined the average genome-wide nucleotide diversity (π) within the larger panel and within the core panel of the nine workhorse test strains [19]. We find that the estimated level of genetic diversity among these *Caenorhabditis* species (within *C. elegans* (1.2e − 3), *C. briggsae* (7.5e − 3), and *C. tropicalis* strains (2.6e − 3)) is higher than that within human populations [20–24] and comparable to that found in mouse populations [25, 26].

In summary, considerable genetic diversity is represented within the core group of *C. elegans* test strains, and this diversity can match, or surpass, the genetic diversity within mouse and human populations. As such, the CITP workhorse screening panel is optimized to facilitate identification of lifespan-extending chemicals enriched for function in conserved processes shared across genetic backgrounds.

#### Accomplishing reproducible survival outcomes is a distinction of CITP efforts

During CITP year 1, the three CITP laboratories worked to accomplish protocol matching, including coordinated purchase and use of same lot number reagents (particularly important for agar), the use of common humidity-controlled incubators for all survival assays (20 °C with 80% humidity, Biological Incubator Model I-36NL, Percival Scientific), and synchronization of autoclaving protocols. The teams agreed on common criteria for scoring animals as dead to finally eliminate variation in survival assay outcomes among labs (see discussion below). To this day, however, the effort requires weekly communication among the CITP site scientists to keep experiments and protocols strictly coordinated. To ensure reproducibility, even very simple assays such as survival of worms on agar in the presence of bacterial food and test compound, can become at least as complex as omic studies.

The CITP adheres strictly to a set of standard operating protocols. Our methods were first described in ref [10]. and can be accessed at Protoc. Exchange https://protocolexchange.researchsquare.com/article/nprot-5341/v1. We highlight some details below.

#### *Caenorhabditis* strain handling

We use test stocks for only 2 months before switching to a fresh stock to prevent genetic drift in wild strains during laboratory use. Animals are maintained continuously on *E. coli* OP50-1 for at least three generations after starvation, thawing, and/or bacterial decontamination before being used in CITP longevity assays to limit epigenetic changes that might influence longevity assessments.

#### Survival assays

To assay survival, we first generate synchronized cultures by collecting eggs from 1- to 4-h egg lays from 1- to 2-day-old adults. When the progeny reach day 1 of adult life, we transfer to 35 mm NGM assay plates with or without intervention, with 50 worms placed on each of at least three individual plates (technical replicates). We consider this as a single trial or biological replicate. We transfer animals to fresh pre-warmed plates with or without intervention tightly adhering to a common CITP transfer schedule. Plates include a low dose of FUdR (51 μM) to inhibit reproduction and prevent progeny from over-running the test population. Because of the potential of FuDR to influence longevity outcomes [27–30], we retest longevity promoting compounds in the absence of FuDR as a facet of phase 4 testing.

The CITP most commonly scores survival manually, with any indication of animal response to prodding with a platinum wire as a criterion for viability. We score alive, dead, lost, bagged (extensive internal egg/progeny retention), or extruded intestine. Bagged, lost, and extruded are “censor” categories in which animals are thought to have died of causes other than aging. “Lost” worms may be missing, burrowed, or attached to plate walls. If bacterial contamination is observed, we score all animals on that plate as lost.

**Fig. 4:**
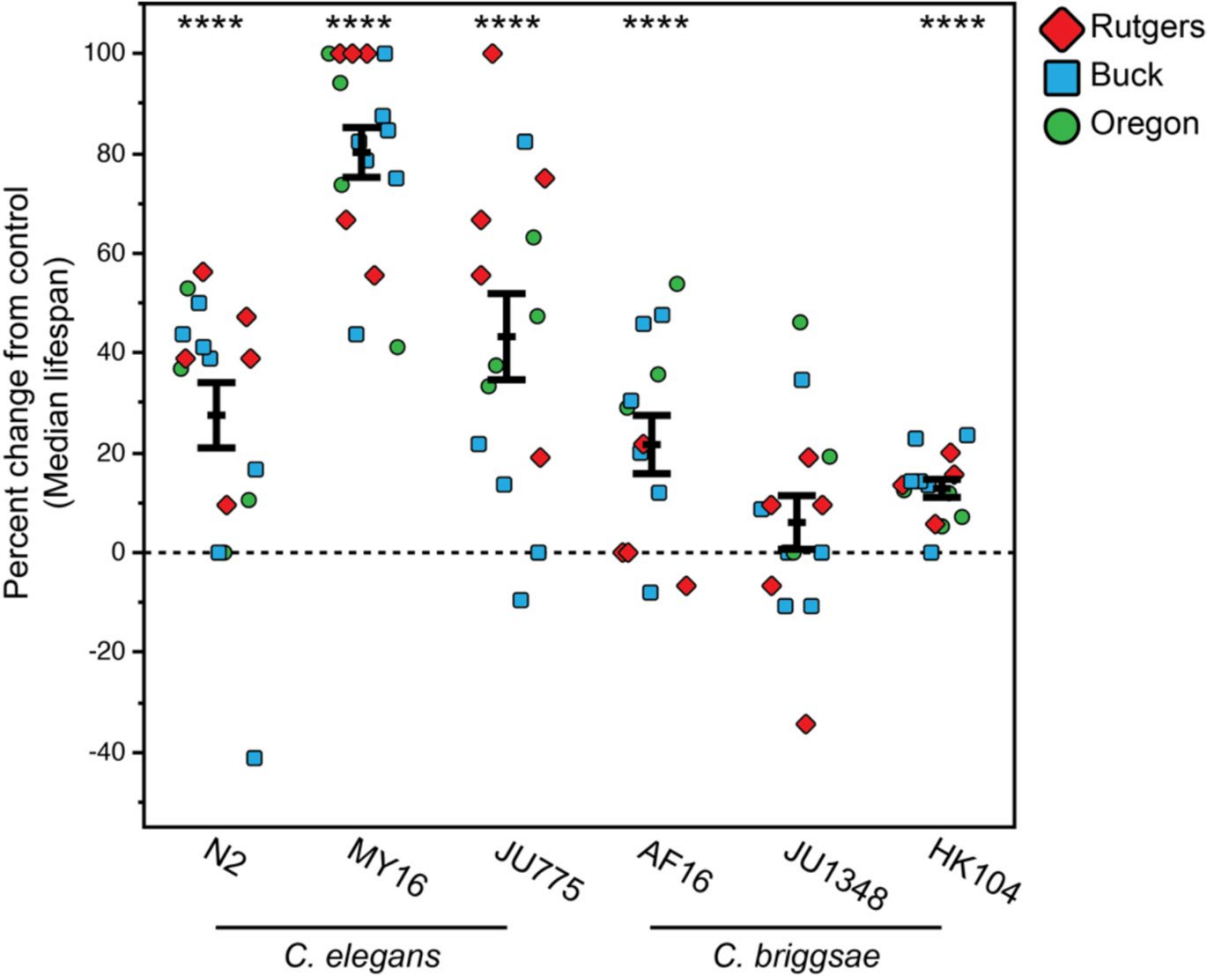
Thioflavin T increases the median lifespan of 3/3 *C. elegans* and 2/3 *C. briggsae* strains. Adapted from Lucanic et al., [10]. Each point represents the percent differences in median lifespan from an individual replicate (treated with 50μM thioflavin T relative to the specific untreated control conducted in the same trial. The bars represent the mean ± the standard error of the mean. Trials were completed at the three CITP testing sites (blue square, Buck Institute; green circle, Oregon; and red diamond, Rutgers). Asterisks represent *p*-values from the CPH model such that ****p < 0.001, ***p < 0.001, **p < 0.01, and *p < 0.05

For publication, we compare survival curves with or without intervention in multiple replicates across labs. We include graphs of the difference in median lifespan between treatment and control for multiple replicates across the CITP test strains as a visually informative representation of how potent a longevity outcome is (example in Fig. 4). Statistical analysis is described below.

#### Data collection, curation, and accessibility provided by the CITP data coordinating center operations

The CITP Data Coordinating Center (DCC) provides essential support for data collection, curation, and storage across all three CITP laboratories.

In addition to careful attention to consistent experimental protocols as a key tool for reproducible science, we quickly discovered that a focus on data collection and curation was also critical. Early on, we discovered that different labs recorded the day of death in slightly different ways, which would naturally be a prime driver of among-lab differences. Equally critical for reproducible analysis was the implementation of consistent metadata information management, including seemingly trivial items like capitalization of strain names, date formats, investigator IDs, and more. Small-scale applications of data collection and maintenance via spreadsheets could introduce human error, and our experience quickly drove the development of a database approach, which has undergone several generations of evolution, resulting in a full cloud-based implementation. This is now coupled to a full open-science base analytical pipeline.

Early on, we established access and data collection forms for all research technicians that served to preserve data quality during collection and transmission and to streamline lifespan analysis pipelines: (1) centralized data control and entry allows real-time verification and validation of metadata as well as out-of-range detection on data input and (2) the ability to immediately map new data into our analysis pipelines enables any unexpected experimental outcomes to be detected and looped back into a quality control discussion with the experimental research team. This approach greatly reduces the need for QC editing of the data before final analysis and has allowed us to quickly address any experimental issues that may arise from unseen circumstances, such as variation in thermal control of incubators. The centralized CITP data approach enhances reproducible science by creating an easy path for an identical analytical framework to be applied to every dataset. CITP has published analysis scripts in the hope of aiding outside researchers in implementing similar methods in their own studies. We conduct all statistical analyses on site at the DCC, using well-defined and verified scripts based in the R statistical framework with which we calculate the effects of compounds on longevity using a mixed-model approach, both with a general linear model (GLM) and Cox proportional-hazards models with random effects (ref [10]., Supplementary Software 2).

The CITP Data Portal on the CITP website provides public access to all lifespan data published by the CITP to date. Pulldown menus include lifespan datasets, summary statistics, and lists of tested compounds with searchable data for each strain, from locomotory assessments to transcriptomics. Data can be identified by publication, compound, or strain, with survival plots for specific queries generated. All downloads are provided in a standardized format for public data analysis. Moving forward, CITP plans to leverage our shared experimental protocols to enhance inferences across disparate phenotypes, from locomotory healthspan to transcript analysis via RNAseq, as well as to expand our survivorship analysis to include more sophisticated models of variable mortality rates. In sum, the public CITP Data Portal lays out data and tools for anyone with Internet access to allow mining the data we produce, which we expect will expand the impact of our massive data accumulation: https://citpaging.org/portal. The public and comprehensive nature of these datasets also lay the groundwork for educational opportunities, which we are in the process of nurturing.

#### Reproducibility assessment is a core practice of CITP: CITP always examines the variation in experimental outcome from multiple trials across three laboratory sites

At the time of CITP inception, a crescendo of commentary focused on a reproducibility crisis in science, a significant concern for the pharmaceutical industry [31–33]. In the invertebrate aging field, controversies over whether intervention outcomes extended, or did not extend, lifespan raged [34–40]. Recognizing the critical importance of reliable data in the aging field, part of the original NIA mandate for the CITP identification of novel anti-aging interventions was that longevity intervention outcomes should be reproduced at distinct sites.

Indeed, CITP data analysis is unique in the invertebrate aging field in that we assess both the effects of compound interventions on individual longevity, and we determine how the variation among several potential sources of that variation is partitioned for interventions that successfully move through the CITP pipeline. Potential sources of variation we have considered are laboratory, species, strain, experimenter, trial, and culture plate. We have also examined possible interactions between laboratory-species and laboratory-strain. This analysis of variation is only possible because of shared experimental approaches and replication across all phases of the work, particularly across strains.

For survival data analysis, we use a mixed-model approach in which “variance-generating” factors are treated as random effects and compounds are treated as fixed effects. R script VarComps.R (ref [10].; Supplemental Software 1) computes the variance component estimates for reproducibility across each of the datasets using hierarchical nested general linear models under a variety of possible error distributions. We examine longevity data for reproducibility at three primary levels: variability in outcomes among labs, variability of outcomes within labs, and sensitivity of outcomes to genetic variation within and among species. Partitioning variation in longevity outcomes to different potential sources of variation in a hierarchical manner using the GLM model has proved quite informative [10].

#### Monitoring variation among sites shows that study outcomes are tightly reproduced across labs

As noted above, monumental effort was required to bring three different labs to follow the exact protocols for survival studies [41]. In our initial publication partitioning variation for longevity data of 10 compounds in a representative six strains across two species we observed, on average, no differences among labs [10]. We have continued to monitor sources of variance for our studies and have maintained high degree of reproducibility among labs (< 6% of total variance).

**Fig. 5:**
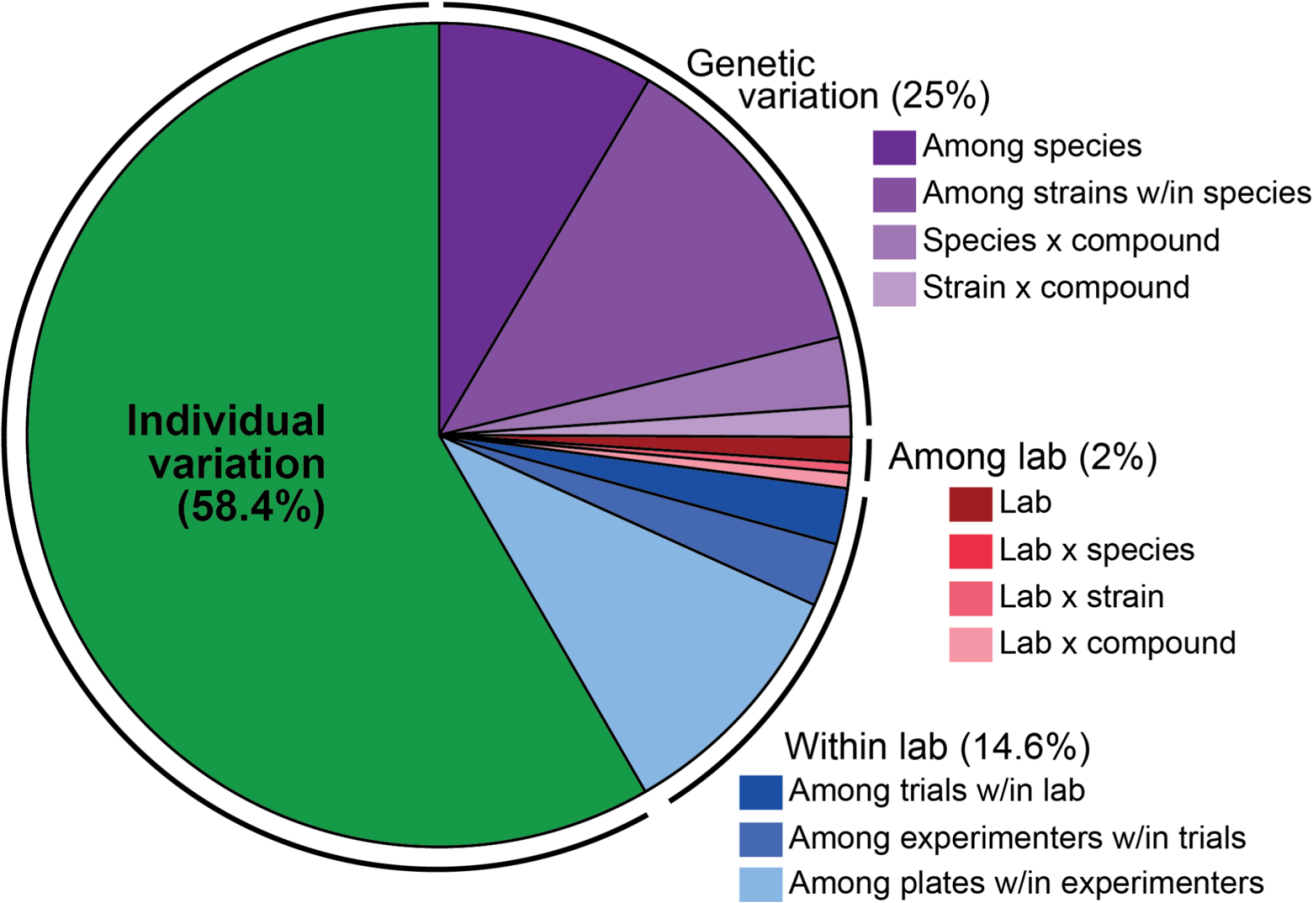
Representation of the sources of variation for longevity across within and among labs for all published CITP manual compound data to date. We considered *n* = 23,1367 individuals across 5436 plates and 150 trials. Variance components were estimated as a randomized block design using a restricted maximum likelihood (REML) general linear model with the *lme4* package (v1.1–35.5) in R (v4.3.3). All factors were treated as random effects. The pie chart shows the total percent variance attributable to various sources—genetic variation (shades of purple): among species (8.5%), among strains within species (12.7%), species by compound (2.7%), and strain by compound (1.2%); among lab variation (shades of red); among labs (1%), lab by species (0%), lab by strain (0.4%), and lab by compound (0.6%); within lab variation (shades of blue); among trials within lab (2.2%); among experimenters within trials (2.5%); among plates within experimenters (9.9%); and individual variation. The percentages listed on the figure are the sum of all categories listed. Of note, there is very little variation attributed to “among lab” differences, supporting strong reproducibility among the CITP labs. At the same time, within each of the 3 CITP labs, we record ~ 14% variance in outcomes that may be attributed to stochastic components that influence outcomes in all labs

Here, we summarize our combined findings based on an updated calculation of all CITP publications to date but point out that the conclusions for individual datasets reflect the overview summary (summarized in Fig. 5; reproduced from data in refs [10, 42–45],, https://doi.org/10.7554/eLife.104375.1). First, it is highly gratifying that we find virtually no systematic difference in outcomes among the three CITP labs, with 2% of total variation in intervention trials attributed to among-lab differences. The high reproducibility among labs underscores that strict adherence to CITP protocols enables us to reproduce outcomes at distinct geographical sites. At the same time, we find it absolutely striking that for *trials within each of the three labs*, we record nearly the same among-trial differences, which account for 15% of the total variation in individual lifespan observed in our studies (most of this variation can be attributed to among-plate differences). In other words, most “error” is generated by undetermined day-to-day differences in conducting an experiment in any lab, not by systematic differences within a given lab. Genetic differences account for most of the variation in these intervention studies (25%). The large amount of residual variation in longevity (59%) is consistent with longevity being an inherently variable trait (this is a necessary consequence of the general shape of a survivorship curve, as individuals tend to not all die on the same day). It should come as no surprise to researchers in the aging field that longevity is a noisy and complex phenotype by its very nature, but CITP has placed definitive quantitation on this truism. We recommend that aging studies conduct multiple, temporally separated replicates to robustly account for this inherent variation.

In summary, CITP documents a reproducible degree of variation in outcome that transpires similarly at each experimental site. As a team, we produce highly reproducible outcomes, but, despite Herculean efforts toward executing the same experiment at each site, there are still factors that can change the longevity trajectories to contribute roughly on the order of 17% of total variance. Factors that might confer variance include inherently stochastic underlying biology of aging [46] or uncontrollable daily fluctuations in microenvironment (for example, atmospheric pressure, temperature change when incubators are opened, intermittent light exposure, etc.) and elements we cannot easily measure, or even imagine. Our documentation of systematic variation among trials underscores the importance of conducting multiple replicates for survival studies, a point that has been well argued previously in the literature [47].

### Integration of new technologies: automated lifespan machines

As noted above, the workflow of the CITP has shifted in task emphasis since its inception. Initially, CITP focus was on meeting high standards of reproducibility and on defining the landscape of how interventions that had been reported to extend lifespan in *C. elegans* reference strain N2 or mouse intersected with efficacy of these compounds across diverse genetic backgrounds as determined by CITP [10]. As the CITP program matured, we maintained focus on reproducibility but adapted to the often-delivered suggestion to increase intervention testing throughput. A component of this effort was the consideration of technologies that might speed our longevity assays.

To this end, we tested and implemented survival analyses with the automated lifespan machine (ALM) [48, 49] developed by Stroustrup et al. [12]. On the ALM, culture plates are placed over a flatbed scanner, and individual animal positions are photographically captured every hour. Software that compares successive images defines whether an animal has moved (alive) or not (dead). During this effort, we introduced several modifications that enhanced ease of lab use of ALMs, which included streamlined physical conversion of scanners into ALMs, engineering of a custom-designed plate holder mat that anchors culture plates over the scanner bed, and changes in scanner cooling strategies [48].

We appreciated clear advantages of ALM use, which include (1) low investigator labor investment during the survival study (plates are put onto the scanners at the start of the study and are not handled for the remainder of the experiment); (2) high scalability such that we could simultaneously run on the order of 20 scanners per lab with ease; and (3) frequent data collection that produces high-resolution survival curves (this is to say data points are recorded every hour rather than every other day). We found this high sampling feature enabled our Data Center team to assess survival curve shapes to the degree we could derive enhanced accuracy of mathematical description of the relationship of age and mortality (compared to the commonly used Gompertz equation). CITP will engage these more precise models in the future to address whether an intervention delays the onset of aging or changes the trajectory of aging itself, among other things (Phillips, *in prep*). Quite important to the CITP mission, we found that ALM-reported survival data were reproducible from lab to lab [49].

We also noted some disadvantages. First, the ALM protocol uses a single compound application to plates, administered at the start of the study, and does not allow for providing fresh intervention exposure during the survival analysis as is typical for manual survival study plate changes. By the same token, ensuring adequate food availability and oxygen availability for the duration of the study becomes a factor. Second, light exposure could photobleach or otherwise change the chemical integrity of the test intervention. For example, we found that thioflavin T (ThioT) was photo-sensitive, such that the robust pro-longevity effect that we documented for ThioT [10] was not observed on the ALM scanners—the addition of a blue light filter to the ALM, however, restored the beneficial effects of ThioT [49]. Likewise, for reasons that we have not determined, alpha-ketoglutarate extended median lifespan in manual survival assays [10], but not under ALM assessment [49]. These observations underscore a theme that emerges strongly from CITP work—context—and the assay conditions selected are highly impactful on study conclusions [49].

Finally, at the time we most heavily used the ALMs, we found we needed to check every video to confirm that the computer called the same death events that a human investigator would call—this post-experiment storyboarding effort added considerable labor to the overall analysis, in part negating the benefits of low investigator investment during the study. Enhanced applications for required computation and scoring have been more recently addressed by the Stroustrup lab, extending to include detailed assessments on individual animals [50, 51], but, in the revised CITP workflow, we returned to manual survival assays as our main component for intervention evaluation. As noted above, we now perform ALM survival assays for compounds that confer ≥ 20% median survival increase in multiple strains, and we extract insights on how interventions either delay the onset of aging or change aging itself thanks to the high-resolution aspect of ALM data (Phillips, in preparation).

### Transcriptomics, clocks, and lifespan extension mechanisms

CITP recently introduced determination of whole animal transcriptomics with, and without, intervention for selected compounds (https://doi.org/10.7554/eLife.104375.1). Although analysis of transcriptional response to intervention treatment is a new addition to the CITP repertoire, it is already clear how the data generated from such efforts/evaluation can inform on mechanism and help to prioritize an intervention for translational testing.

For example, we found that all-trans-retinoic acid (atRA), a signaling ligand clinically used for treatment of acne, skin photoaging, and acute promyelocytic leukemia, extended median survival for the three representative *C. elegans* test strains on the order of 20–30%, with modest enhancement of swim behavior at middle age (https://doi.org/10.7554/eLife.104375.1). Guided by transcriptomics data, we utilized genetic approaches to document requirements of kinases AKT-1 and AKT-2, their conserved target transcription factors Nrf2/SKN-1 and HSF1/HSF-1, and the AAK-2 catalytic subunit of AMPK as required for longevity extension by atRA. CITP data thus support that the all-trans-retinoic acid pathway is an ancient metabolic regulatory system that can modulate lifespan via multiple longevity pathways, which invites increased attention to longevity and health testing of atRA in higher organisms.

Overall, as intervention-specific RNAseq data accumulate in the CITP database, we anticipate logical extension into generation of strongly data-anchored transcriptomics aging clocks. Indeed, the CITP transcriptomics data are likely to become a key resource in the aging field in that all of our data are collected using rigidly standardized protocols and outcomes are reproduced, which is not the case for the studies anchoring current meta-analyses of transcriptomic data in the field. The CITP transcriptomics database can thus clearly make a broad contribution to the evaluation of how interventions generally engage gene expression networks over adult age. Transcriptomics data can also inspire consideration for combinatorial therapy designed to engage multiple longevity pathways for optimal health benefit [52]. Finally, transcriptomics on responder vs. non-responder *Caenorhabditis* variants in CITP should also inform on molecular conditions for intervention efficacy over populations.

### CITP consideration of healthspan metrics

A common consensus in the aging field is that maximally maintained functionality over lifetime, i.e., a long healthspan, should be a critical goal for therapeutic intervention [53]. The CITP has investigated how chemical interventions influence multiple health metrics, with limited focus.

#### Locomotory decline

This is a feature of aging across the animal kingdom, and mobility is an often-cited indicator of human life quality [54]. The CITP quantitates *Caenorhabditis* swim locomotion as an indicator of locomotory vigor in older age. We chose swim behavior (thrashing in liquid) for focus over simple plate crawling movement because the swim behavior can be analyzed for multiple measurement parameters scorable over a greater dynamic range and for a longer period of adult life than with crawling. For example, good dynamic ranges are found in the wave initiation rate (similar to head thrashes per unit time individuals score manually) and activity index (which measures the area in pixels that the body of the animal covers (“paints”) in two strokes). CITP uses a computational program called CeleST (a *C. elegans* Swim Test) for analysis of swim behavior, which can score multiple swimmers simultaneously for eight distinct measurement parameters, and offers fully automated tracking and statistical analysis programs [11, 55]. Our statistical team at the CITP Data Center used linear discriminate analysis to weight and combine the eight individual measurements into a single multivariate composite measure of overall swimming ability [45]; this mobility measure, reminiscent of human “frailty index” [56], is currently measured on most potent CITP anti-aging interventions, in phase 4 analyses of compound effects on mobility decline.

On the whole, CITP assessments of locomotory healthspan consequent to intervention treatment generally support that maintained swim vigor is not well correlated with enhanced longevity [45]. On the plus side, we have observed that locomotory healthspan extension can be an outcome of a compound intervention, even in the absence of lifespan extension. For example, CITP found that the anti-diabetes drug metformin increases median survival in three *C. elegans* strains, but metformin fails to increase *C. briggsae* median survival. By contrast, metformin prolongs the period of youthful vigor in two of three *C. briggsae* test strains as well as in all *C. elegans* strains [14]. We reported a similar outcome for nordihydroguaiaretic acid (NGDA) in *C. briggsae *[45]. On the other hand, some interventions that extend lifespan in CITP do not markedly alter the decline in swim vigor.

#### Oxidative stress and heat stress

CITP used automated lifespan machines to assess the impact of representative longevity-promoting interventions on oxidative stress (paraquat exposure) and thermotolerance (survival at 32 °C). We tested representative pro-longevity compounds (candidate dietary restriction mimetic NP1, oxidative stress pathway-implicated propyl gallate, and red wine component resveratrol [45], as well as anti-oxidant NDGA and green tea extract, GTE) [13], and we compared the oxidative and thermal stress outcomes with locomotory outcomes. Overall, we find that swimming ability and oxidative stress resistance do not correlate with longevity; thermotolerance correlated better with longevity [45] but could be quite variable [45]. Thus, although we were able to use ALMs to assess animal movement in oxidative and thermal stress studies, the experimental effort required, coupled with the generally poor correlations with longevity, led us to adjust our workflow such that health assessments (primarily locomotion) were moved to phase 4 for interventions that conferred ≥ 20% median lifespan extension.

Separation of lifespan and healthspan outcomes is not uncommon in the aging field [57]. Still, a paucity of intervention screens feature a dedicated focus on healthspan and promising vigor-promoting interventions may be sidelined because of the lack of longevity effect. Given that healthspan-promoting compounds hold huge potential for improving adult life quality [58], focus on healthspan endpoints might be encouraged in the future.

As the CITP increasingly accumulates RNAseq data during adult life interventions, novel health indicators that strongly correlate with longevity (for example, aging clock signatures) may anchor screens for interventions that extend the youthful physicality period of life.

### Summary of CITP contributions to the aging field 2013–2024

The CITP initiated work in 2013. We dedicated nearly 2 years of effort to optimizing reproducibility and published on longevity data for 22 reference strains in 2017 [10].

Initial testing focused on high “public” profile compounds such as resveratrol [10], rapamycin [13], and Metformin [14] as well as promising compounds from literature or compounds already known to the CITP team. Over time, we (1) established the route for soliciting and selecting compounds suggested by the public; (2) considered the growing body of structural and functional data for chemicals and processes that influence longevity and health predictions (for example, 10.7554/eLife.104375); and (3) conducted chemical library screens in house (Lithgow, *in preparation*). Currently, the CITP Steering Committee discusses and approves all interventions that enter the pipeline, and CITP has a strong focus on novel compound identification strategies.

As of December 2024, CITP tested a total of 77 compounds (Tables 1 and 2) and conducted a total of 730,731 animal assays over 891 trials. We identified 27 compounds (not including those currently in testing) that exhibit statistically significant lifespan extension in at least one strain. Thirteen of these confer a ≥ 20% increase in median lifespan in at least 2 strains, with 12 reproducibly and robustly extending lifespan across multiple strains and labs.

**Table 1:** Summary of all compounds tested by the CITP to date and their effect size

Hits			Non-hits			
≥ 20%	≥ 10 to < 20%	< 10%	No/negative effect			Currently in testing
All-trans retinoic acid	Alpha lipoic acid	17-Alpha estradiol	Acarbose	Decitabine	LY2603618*	2-Deoxyglucose
Alpha ketoglutarate	Berberine	Obeticholic acid	AZD7545*	Dexamethasone	Meclizine*	Azure b
Bromopyruvate*	Bromocriptine	Ritonavir	Aldosterone	Dicoumarol*	Metoprolol	Crocetin
Gold sodium thiomalate*	Coumarin 478*		Aloin*	Diuron	Nateglinide	Dynasore
Green tea extract	Echinatin*		Alpha mangostin*	Emapunil*	Pioglitazone	Ethosuximide
Metformin	Fisetin		Arecoline	Erlotinib	PF477736*	Nicotinamide
NDGA	Fluspirilene*		Aspirin	Everolimus	Quercetin	Nicotinamide Mononucleotide
NP1	Rapamycin		Bakuchiol	Fumagillol	Schaftoside*	Nicotinamide riboside
Propranolol†	Sitagliptin		Beta GPA	Gamma linolenic acid*	Tamibarotene	Nicotinic acid
Propyl gallate			Bortezomib	Gefitinib	Temsirolimus	ZGN-1062
Resveratrol			Cinnarizine*	Glipizide	Thalidomide	
Sulforaphane			Curcumin	Lactulose*	Valproic acid	
Thioflavin t			Dapagliflozin	Imatinib		
Urolithin a*†			Dasatinib	Levetiracetam*		

≥ 20%, ≥ 10 to < 20%, and < 10% categories indicate the maximal percent change in median lifespan observed in at least one strain. All ≥ 20% compounds were tested in at least six strains from two *Caenorhabditis* species across all three laboratories (^†^with the exception of propranolol and urolithin a which were not tested in the full CITP) and had significant lifespan extension in a minimum of two strains. Asterisks represent compounds that are complete but pending publication, and currently, in-testing compounds are still in the CITP pipeline

**Table 2:**
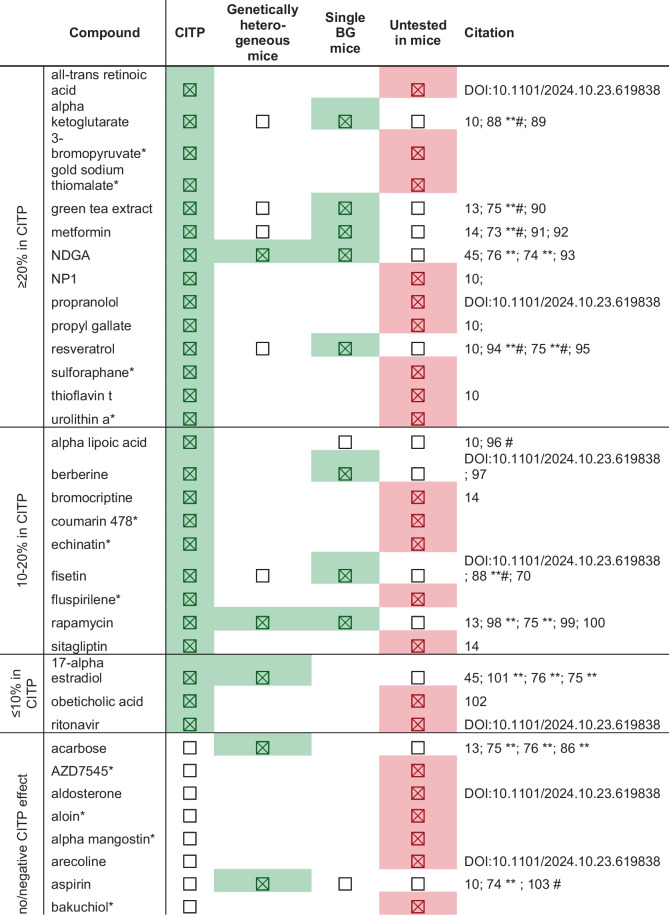

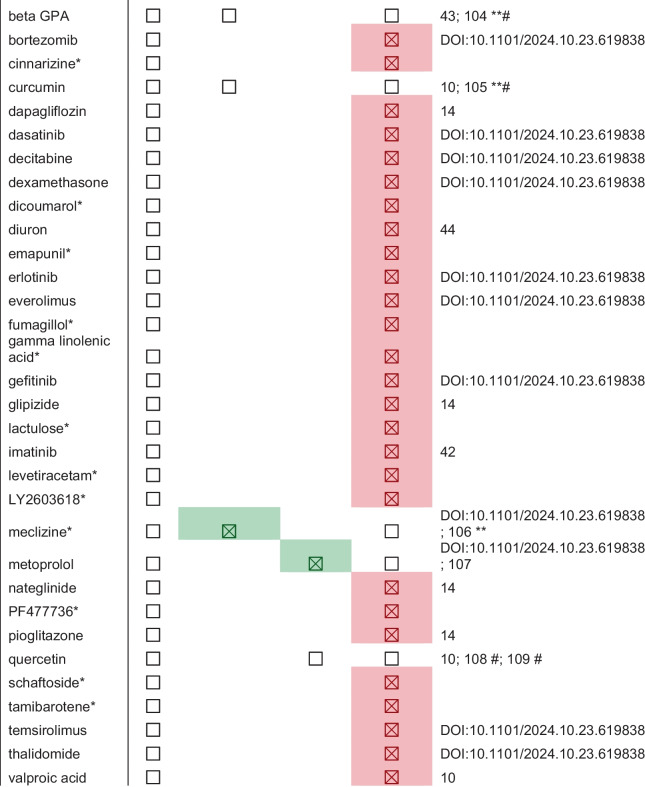
Summary of all completed compounds tested by the CITP and comparisons to mice studies in the literature

For the CITP, heterogeneous and non-heterogeneous mice columns: a green checked box indicates a positive lifespan hit for that category, with a hit defined as any statistically significant lifespan effect, regardless of sex. An unchecked box indicates that the compound was studied for lifespan effects with neutral or negative outcomes. Empty cells indicate compounds for which we were unable to find published studies within the context of our search criteria. For the untested in mice column: red checked boxes represent compounds that were tested by the CITP but have not yet been tested in mice; white unchecked boxes denote compounds that have been tested in mice. To reference the literature, we first searched for compounds using the DrugAge database (https://genomics.senescence.info/drugs/browse.php; accessed January 2024) and then used the search terms “compound name mice lifespan” on PubMed and Google Scholar, viewing the first two pages of results. All compounds were also cross referenced with the ITP data portal (https://phenome.jax.org/projects/ITP1). ITP results are indicated with a ** and mouse studies with mixed outcomes are denoted with a # in the citation column

Here, we briefly review some CITP highlight contributions to the field, several of which extend beyond the simple testing of candidate geroprotectant compounds.

#### –*Weestablished protocols for reproducible assessment of adult survival using 22 reference strains that spanned three Caenorhabditis species *[10]

Data enabled us to show that the CITP labs could coordinate efforts to conduct survival assays with outcomes for which minimal variance could be attributed to “among lab” differences. At the same time, we documented ~ 15% of variance of experimental outcome that “reproducibly” transpired within each of the three CITP labs, possibly attributed to unknown factors that influenced outcomes (for example, see baseline longevity for 22 CITP strains across the 3 CITP labs, Fig. 2, Table 1 in Ref.) [10]. Most of the variation in CITP survival outcome can be attributed to genetic differences and individual differences [10]. Data speak to the inherently variable nature of the longevity phenotype and may explain some of the among-lab controversies on intervention outcomes in the aging field [41]. Data also underscore the critical importance of repeat trials.

Over time, the CITP program has been highly cited as a model for considerations of reproducibility challenges in science [59–64].

#### *–We established a public nomination mechanism for interventions and added web interface to support the application process and progress dissemination*

NIA Scientific Officers Dr. Max Guo and Dr. Tiziana Cogliati established an annual call for compound nomination, provided links to application forms (https://www.nia.nih.gov/research/dab/caenorhabditis-intervention-testing-program-citp/citp-intervention-nomination-guide), recruited an expert Access Panel that reviews compound proposals in NIH-grant-review style, and arranged for final approval by the CITP Steering Committee. Approved compounds enter the CITP pipeline as a collaboration with nominators, and data are published regardless of outcome. It is both exciting and gratifying that we have found that some of the nominated compounds confer reproducible longevity. For example, one compound that has emerged via public submission is the broccoli component sulforaphane. We find that sulforaphane can confer significant lifespan (~ 50% median lifespan extension in *C. elegans* N2) and mobility healthspan extension in the *C. elegans* genus. Our transcriptomics studies reveal a transcriptional signature response to sulforaphane that reflects increased expression of detoxification gene batteries (Sedore, *in preparation*).

#### *–We developed a CITP Data Coordination Center that serves all project needs in data accumulation, storage, and statistical analysis*

Intervention survival data for each compound can be easily accessed on our data portal (CITPaging.org). The DCC also manages locomotory (CeleST data and statistics), transcriptomics, and mortality curve analyses. Our centralized database facilitates data collection, quality control, and curation to enable rapid analysis by CITP and by interested outside parties who can access the raw data and the R scripts we use for analysis. The portal for public applications for CITP testing is housed on the NIH CITP website https://www.nia.nih.gov/research/dab/caenorhabditis-intervention-testing-program-citp/citp-intervention-nomination-guide.

#### *–We contributed to technology development/utilization in the field*

CITP published extensively detailed protocols and commentary on considerations for best practices toward accomplishing reproducible longevity data [10, 41]. We developed, and made available, R scripts that can be used for survival analysis, attribution of variance, locomotion quantitation, and, more recently, mortality curve analyses. The CITP published user-friendly modifications to automated lifespan machines [12] and discussed considerations their use [49, 48], with the hope that sharing lessons learned and the modifications we implemented will help users adapt ALM technology into their labs.

#### *–We identified 12 compounds that reproducibly extend median survival*

≥ *20% in at least 1 strain and are efficacious across multiple backgrounds* (Table 1). The majority of these compounds are most efficacious in the *C. elegans* strain set, which might be expected since interventions are commonly selected as plausible candidates due to prior positive influence on *C. elegans* N2 longevity. The positive outcome in 12 of 69 (excluding several promising compounds still in the pipeline) tests constitutes an ~ 17% success rate for CITP testing over time.

CITP makes a point of emphasizing that negative outcomes should not be interpreted to mean that the compound fails to extend lifespan. Rather, the conclusion to be drawn is that testing of that compound at three sites across a genetically diverse test set and under the CITP standard operating protocols was not effective in promoting enhanced adult survival.

Most interventions work in multiple, but not all, strain backgrounds, an observation that might be attributable to differences in compound uptake or metabolism, genetic background, or test conditions, to name just a few of many possible explanations. As our testing efforts progress, CITP data on transcriptomic and metabolomic responses to compounds across genetically diverse and differentially responding test strains may address potential bases for why certain compounds affect some strains and species differently.

*–We reported on interventions that enjoy high profile attention in both the public domain and in the scientific literature*.Resveratrol, the renowned red wine component that engages sirtuin activity, extended median lifespan in *C. elegans* strains (especially JU775, which had ~ 40% relative median extension ) [10]. α-Ketoglutarate, the TCA cycle intermediate that serves as a biosynthetic precursor for glutamate and glutamine, may inhibit TOR [65] and is commonly found in “health food” stores, extended median lifespan > 20% in all three *C. elegans* test strains, with a striking > 50% increase in strain MY16 [10]. As mentioned above, broccoli component and health supplement sulforaphane was a publicly nominated compound that is highly efficacious (~ 50% median lifespan increase) in *C. elegans* strains (Sedore, *in preparation*).

CITP analysis of green tea extract also supported lifespan (significant lifespan extension in five strains across *C. elegans* and *C. briggsae*) and locomotory healthspan extension (modest enhancement in multiple test strains) [13]. Given the complexity and variability in extract components, however, the CITP plans to focus on testing interventions with concrete and invariant chemical makeup moving forward.

The biguanide metformin is broadly prescribed to diabetic patients, and data have suggested health benefits that extend beyond balancing of sugar metabolism to include limitations on inflammation and cell senescence. Indeed, exciting trials designed to address metformin as a human anti-aging intervention loom (see the TAME project (Targeting Aging with Metformin) [66]. To model how metformin influences heath and aging outcomes in a genetically diverse population with sequenced genomes, we assayed metformin treatment of the nine CITP test strains [14]. Overall, we documented an impressive and broad health benefit: metformin increases median survival in three *C. elegans* strains and enhances locomotory capabilities in all three *C. elegans* and two *C. briggsae* strains, placing metformin with some of the most potent anti-aging compounds CITP has measured. CITP data thus underscore the potential of metformin to broadly improve older age health and survival in a genetically diverse population.

That said, it is important to point out that metformin was not uniformly efficacious across the *Caenorhabditis* test set—*C. briggsae* strains were not long-lived, and metformin was modestly deleterious when fed to *C. tropicalis*. The metformin/genetic-heterogeneity interface emphasizes that a “one size fits all” outcome might not be anticipated for human metformin intervention and underscores that detailed attention to individual genetic backgrounds might be needed to accurately assess proposed metformin benefits (a consideration for environmental factors as well); a personalized medicine component may be required to maximize health outcomes across a population for this intervention.

CITP did not find strong positive outcomes for some other popular treatments: aspirin, quercetin, and curcumin did not confer longevity benefit [10] despite previously recorded beneficial effects for testing under a different protocol.

**Fig. 6:**
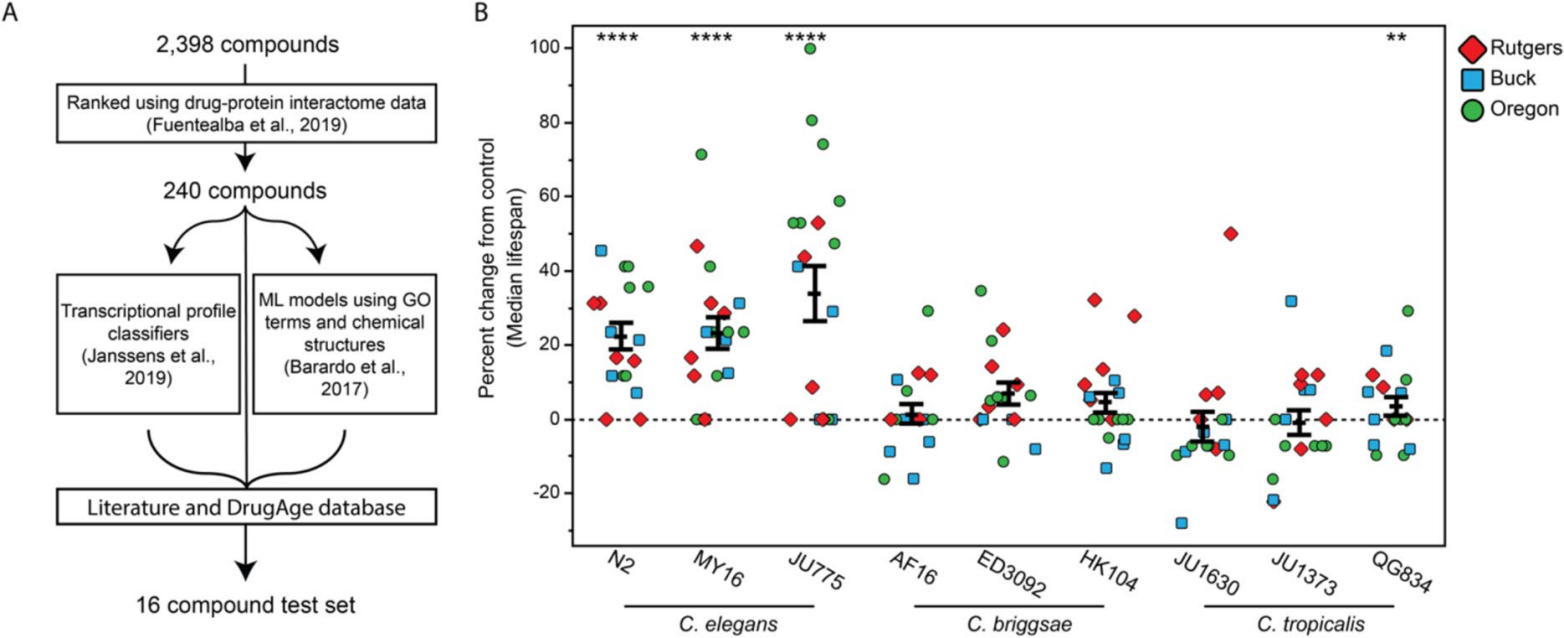
The use of computational predictions for lifespan extending compounds in the literature identifies all-trans retinoic acid as a pro-longevity compound. Adapted from Banse et al. [13] (https://doi.org/10.7554/eLife.104375.1). **A** A summary of how compounds were selected for testing by the CITP. The top 10% of predicted hits from Fuentealba et al. [15] were cross referenced with compounds that appeared in the top 10% of Barardo et al. [16] or Janssens et al. [68] or had been shown to work in other model organisms via the DrugAge database [87]. **B** 150 µM all-trans retinoic acid robustly and reproducibly extends lifespan of *C. elegans* N2, MY16, and JU775, but not *C. briggsae* AF16, ED3092, and HK104, or *C. tropicalis* JU1630, JU1373, and QG834. Each dot represents the percent difference in median lifespan of an individual ATRA trial plate, as compared to its specific control. Replicates were conducted at the three CITP testing sites (blue square, Buck Institute; green circle, Oregon; red diamond, Rutgers). The bars represent the mean ± the standard error of the mean, and asterisks represent *p*-values from the CPH model such that ****p < 0.0001, ***p < 0.001, **p < 0.01, and *p < 0.05

*–We incorporated state-of-the-art data mining approaches toward the identification of novel successful intervention candidates* (10.7554/eLife.104375). We took advantage of the ever-expanding data available on proposed heath- and life-promoting chemicals [67] to consider compounds for CITP testing (strategy summarized in Fig. 6A). More specifically, we considered the top 10% of candidates from a list of computationally ranked compounds built using known drug-protein interaction [15], eliminated overlap with compounds previously shown to be efficacious in *C. elegans* N2, or to impact bacterial food growth, to emphasize novel discovery, and considered intersection of that revised list with lists for predicted aging effects based on comparative transcriptional responses [68] (all-trans-retinoic acid, arecoline, propranolol, thalidomide) and machine-learning models based on gene ontologies and physical structures [16] (aldosterone, berberine, bortezomib, dasatinib, decitabine, dexamethasone, erlotinib, gefitinib, ritonavir, temsirolimus), or listing in the DrugAge database for lifespan extension in other systems (everolimus [69]; fisetin [70]). Of the candidate list, we found that 5/16 tested compounds (all-trans-retinoic acid, berberine, fisetin, propranolol, and ritonavir) extended lifespan (as an example, see Fig. 6B for all-trans-retinoic acid results), while the others had no effect or were toxic.

The > 30% positive hit rate we found in this study both establishes proof of principle that the computational approach constitutes a promising pipeline strategy and emphasizes how CITP can rapidly generate robust and reproducible data on the growing number of novel candidate compounds that are being published. Expanding similar data mining strategies will enable CITP to identify, and rapidly test for the potential of, novel interventions.

### Perspectives: chronic disease and preventive medicine

The foundations of the use of simple model organisms such as *C. elegans* are twofold. First, *C. elegans* is a highly simplified yet sophisticated animal displaying classic aging functional decline over a short lifespan. Based on the remarkable commonality of core biology across animals, lessons learned in *C. elegans* are likely to be applicable to studying human aging. Indeed, with the exception of cellular senescence, several known hallmarks of aging [71] were discovered in invertebrates. Second, the geroscience world view posits that intervening in aging can have beneficial effects across diverse disease pathologies. Thus, the identification of compounds that postpone aging in *Caenorhabditis* strains can be the beginning of drug-development pipelines for the prevention of human chronic disease.

The CITP monitors how interventions that result in positive outcomes in the genetically heterogenous *Caenorhabditis* perform in testing in mouse (Table 1). For the 28 CITP compounds that confer statistically significant lifespan extension in some of the test *Caenorhabditis* strains, 9 have been reported to have some pro-longevity effects in mouse (ITP or individual studies); 18 are not published for mouse. Other compounds or related compounds have beneficial effects on functional aging in mouse [72].

Among the 12 CITP compounds that exert ≥ 20% median lifespan extension and are efficacious across multiple test strains, 5 enhance mouse longevity, while testing of the other 7 has not yet been reported. Based on these numbers, at least 40% of CITP positive outcomes are shared with mouse outcomes.

In the mouse Intervention Testing Program (ITP), three geographically separated labs test mice generated from a four-way genetic crossing strategy to introduce genetic heterogeneity [73]. The CITP and ITP are two independent programs such that success of an intervention in nematodes in the CITP does not automatically feed into the ITP pipeline, and vice versa ITP-compounds that are efficacious in the genetically heterogenous UM-HET3 mice are not automatically further explored in the CITP pipeline. However, in the initial CITP years, multiple ITP-tested compounds were evaluated [10] to assess the relationship of findings in the two programs. The overlap in interventions that have been tested in both the CITP and ITP is shown in Table 1.

To date, five compounds have been tested and published by both the CITP and ITP: α-ketoglutarate, GTE, metformin, resveratrol, and NDGA. Of these, NDGA exerts longevity extension in genetically diverse MU-HET3 male ITP mice using standard ITP log rank statistical criteria for longevity assessment [74–76]. Recently, ITP reanalyzed 2002–2022 data using the Gehan-Breslow-Wilcoxon test, which is similar to the log rank test but does not assume proportional hazards over the lifetime and, thus, can better identify interventions that modulate mortality only early in life. In this re-evaluation, metformin and GTE emerge as pro-longevity interventions [77]. The intersection of positive outcomes for 3/5 commonly CITP/ITP-tested interventions suggests the potential for the CITP to identify broadly acting geroprotectant candidates with reasonable probability of success in mammals. This conclusion is tempered by the fact that relatively few compounds have undergone rigorous testing in both *Caenorhabditis* and the lab mouse.

Although encouraging, it always been clear that biological differences between invertebrates and mammals are certain to sideline multiple candidate interventions. On the *Caenorhabditis* side, considerations such as interaction of tested interventions with the bacterial (*E. coli*) food source [78–80], the potential of the interaction of FuDR treatment (to limit offspring production) with tested interventions [27–30], internal physiology and pharmacokinetics impacting intervention persistence, and *Caenorhabditis-*specific biology are all factors that might impede translation. To maximize translation-potential assessment, CITP now tests successful compounds for food and FuDR interactions as a routine component of studies. On the mouse side, limited options for dosing and timing of intervention, specifics of intervention persistence and metabolism in the animal, and artificial, sedentary cage environment can modulate outcome, to name just a few possibilities.

Direct comparison of longevity effects in nematode and mouse should also take into account different longevity metrics in the two species. Studies in mouse and nematode address different aging challenges. Successful interventions in mouse may counter common causes of old-age mouse death in the lab such as cancers [81]. *Caenorhabditis* interventions are unlikely to engage cancer-related molecular mechanisms per se (all *C. elegans* cells are post-mitotic in adulthood). Instead, successful *Caenorhabditis* interventions might primarily target stress pathways or engage hormesis to influence nematode survival. Still, the foundational idea that an intervention that engages a highly conserved pathway can be bioactive in both models has compelling precedent—the condition for success is that the biological activity of any intervention is preserved in the physiological context of each animal.

#### Interventions related to *Caenorhabditis*-identified geroprotectants can confer unexpected benefits to aging mice

Longevity is just one of many valuable translational outcomes of pro-longevity interventions that extend *C. elegans* life. Indeed, implications of the geroscience hypothesis (because aging predisposes to many of the diseases of older age, interventions that target the aging process itself will protect against multiple disorders of older age [82]) may be commonly overlooked in the aging field discourse on intervention value. For example, CITP work on ThioT provides a springboard for considering how intervention efficacy might play out for health promotion in higher models. Thioflavin T is an amyloid-binding reagent that can extend lifespan and slow aggregation toxicity in multiple *C. elegans* models of proteotoxicity, including the expression of human Alzheimer’s disease-implicated neurotoxic peptide Aβ_3–41_[83]. CITP documented that ThioT can enhance survival in three *C. elegans* and two *C. briggsae* CITP test strains, supporting a broadly efficacious intervention capacity over a genetically diverse test set [10]. The mechanism by which *Caenorhabditis* aging health is enhanced might operate via proteostasis modulation as it requires a range of regulators of proteostasis for lifespan extension, although ThioT interactions with bacterial food have also been suggested [84]. Whatever the mechanism of action in *Caenorhabditis*, treatment of older mice with amyloid-binding synthetic molecule 2-hydroxyphenyl-benzoxazole (HBX) (the design of which was based on ThioT) prevented femoral bone loss[72] and protected against ALS pathology [85]. Mouse ITP testing found male median lifespan to be increased by HBX [86]. In other words, a *C. elegans* influenced intervention in mouse conferred multiple positive health outcomes in aging mice.

Why would an intervention that enhanced longevity in diverse *Caenorhabditis* promote bone health and brain health in aging mice? From a geroscience hypothesis perspective, conserved pro-longevity modulators identified in *C. elegans* are predicted to confer unexpected benefits for healthy aging across phyla. A point that may be underappreciated is that invertebrate intervention validation may identify compounds with unexpected benefit in mammalian aging, possibly with segmental or tissue-specific outcomes. CITP data support that attention to such a possibility can play out, at least in aging mice.

Overall, although many compounds are likely to fall out of the invertebrate to mammalian pipeline, those interventions that emerge across models have an enhanced chance of efficacy in humans. Testing against a landscape of genetic heterogeneity reduces concerns regarding genetic background-specific effects and the use of wild-derived strains (i.e., not laboratory adapted) is a powerful argument for high relevance.

The chronic diseases of late life remain the biggest medical, social, and economic challenge of this century. Despite the investment of considerable resources into finding medicines that combat debilitating diseases such as Alzheimer’s and cardiovascular disease, successes are disappointingly few. If indeed aging is a common cause of such diverse conditions, then targeting the aging processes themselves is surely a potential starting point for drug development. Many models are available, including human cell culture, for the discovery of small molecule modulators of aging processes. However, models that encapsulate the entire aging process in an intact living organism are few. The CITP has proven to be an excellent model for the discovery of small drug-like molecules that influence mammalian aging.

Should we expect cures or preventions for chronic disease from the geroscience perspective? Certainly both, but the CITP approach has been to treat animals from early adulthood and is therefore more likely to be a starting point for prevention. One of the challenges of chronic disease therapeutics is the attempt to mitigate symptoms or reverse pathology neglecting etiology. While efforts to reverse or mitigate pathology in diseases such as Alzheimer’s and Parkinson’s must continue, the possibility of prevention may be more realistic. The CITP is well placed to present candidate preventative therapeutic avenues to the biomedical community.

### Summary and future perspectives

In the 13 years since its incipit, the CITP has established powerful experimental and statistical approaches for testing pharmacological interventions over a genetically diverse test set of *Caenorhabditis*, a field-leading model in aging research. A significant proportion of interventions that have emerged from the CITP pipeline can exert benefits in mouse aging, which speaks well to the potential to continue to serve up candidates for pre-clinical and clinical research. As the aging field adopts enhanced detection algorithms and high throughput screens, increasing numbers of candidate geroprotectants will be suggested. In future, we expect an increasing need for rigorously tested compounds to establish priorities for enhanced testing in mammalian models and clinical applications. The CITP program is well poised to meet this challenge.

The evolution of CITP has included workflow refinement to increase throughput, incorporation of intervention-induced transcriptomic signatures to inform on mechanism, development of multiple strategies for identification of novel longevity-promoting compounds, and advances in technological approaches to longevity and locomotory healthspan assessment. Possibly most rewarding has been the opportunity for a group of scientists, working across the country, to combine talents toward the common goal of generating reproducible, high-quality longevity data. We have stumbled upon unexpected insights into the biology of aging on a path that generated friendships as well as intellectual engagements and accomplishments.

Our opportunity to work closely with dedicated NIA staff, including Drs. Max Guo, Ron Kohanski, Viviana Perez Montes, Tiziana Cogliati, and Jennifer Fox, has opened our eyes to the devoted efforts of NIA staff to take on the colossal challenge of improving the health of the aging public. The CITP is proud to be a facet of this effort, and we anticipate moving forward with the momentum we have built to generate highly impactful outcomes in the future.

Happy Anniversary NIA, with gratitude for your tireless efforts, CITP.
